# Effect of Sandpaper Meshes on the Performance of *Tilia* Sp. Self-Repairing Coatings

**DOI:** 10.3390/polym15132835

**Published:** 2023-06-27

**Authors:** Yijuan Chang, Zhihui Wu

**Affiliations:** 1 College of Furnishings and Industrial Design, Nanjing Forestry University, Nanjing 210037, China; 2 Co-Innovation Center of Efficient Processing and Utilization of Forest Resources, Nanjing Forestry University, Nanjing 210037, China

**Keywords:** self-repairing microcapsules, sandpaper mesh, roughness, properties

## Abstract

This study aimed to investigate the impact of different sandpaper sanding meshes on the mechanical and optical properties of microencapsulated *Tilia* sp. film. An orthogonal experiment revealed that sanding between primers had the most significant effect. Furthermore, an independent experiment implied that increasing the mesh size resulted in decreased surface roughness and decreased color difference, elongation at break, and gloss after liquid resistance. In the aging test, the color difference of the paint film increased with the aging time, and the gloss tended to stabilize. Additionally, the anti-aging gloss of 240 mesh sandpaper used between primers remained relatively stable. The paint film sanded with 240 mesh sandpaper between primers displayed small and regular cracks after temperature and UV aging. Overall, the paint film demonstrated good comprehensive performance when sanded with 240 mesh between primers, 240 mesh between primer/topcoat, and 1000 mesh for topcoat. Self-repairing microcapsules showed better repair efficacy on the coating. This study provides a technical reference for the development of self-repairing coatings.

## 1. Introduction

**Figure 1:**
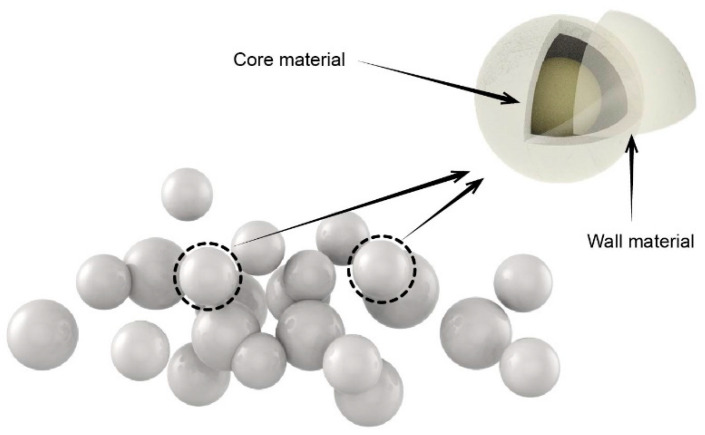
Structure of self-repairing microcapsules.

During extended use, wood experiences microcracks due to its inherent properties of natural dry shrinkage and wet expansion. Wood can absorb moisture and breed bacteria, leading to a decrease in its service life. While coating can provide protection against cracking and decaying of the wood [1,2], it can also result in aging cracking during prolonged use. The self-repairing microcapsule can solve this problem. The self-repairing mechanism of microcapsules involves incorporating healing agents into the target material [3,4]. Add self-repairing microcapsules to wood water-based coatings; when the wood coating experiences external impact, microcracks emerge [5] (Figure 1); the microcapsule wall material ruptures when it reaches the stress trigger point, allowing the core material to flow out and repair the microcracks [6,7,8,9].

In previous studies on self-healing microcapsules, Xu et al. [10] conducted a study on self-repairing microcapsules and coating performance, wherein they synthesized and characterized an epoxy microcapsule, which was encapsulated with urea-formaldehyde. To control the release of self-repairing materials and trigger microcapsules in concrete, they creatively employed ultrasonic as an artificial trigger. Li et al. [11] used melamine resin as the wall material to prepare magnetic carbonyl iron powder (CIP) microcapsules by in situ polymerization. They were mixed with shellac self-healing microcapsules to prepare dual-functional wood coatings, and the effects of different amounts of CIP microcapsules in Dulux waterborne primers on the properties of the primers were studied. The results showed that the core-to-wall ratio had a significant effect on the properties of CIP microcapsules. When the core-to-wall ratio is 0.65:1, the coverage rate of microcapsules reaches 57.7%. Aside from the reparative characteristics of the paint film, the roughness and adhesion of the film are equally crucial. Akinci et al. [12] evaluated the adhesion behavior and surface properties of coatings on molds and polyurethane materials used in the production of automotive seats. They coated four different types of polymers on aluminum molds and analyzed them based on various parameters, such as contact angle, surface roughness, coating thickness, adhesion, cross-cutting, and SEM. The perfluoroalkoxy coating had the lowest surface roughness, while the ethylene-tetrafluoroethylene coating had the highest. The results demonstrated that adhesion increased with an increase in surface roughness. In another study, Salvadori et al. [13] studied the surface roughness of DLC-coated substrates as a function of film thickness. For substrates with original roughness of 393 and 278 nm, the coating roughness increased with the increase in DLC thickness, reached a maximum, and then decreased. For substrates with an original roughness of around 4 nm, the coating roughness showed no systematic decrease or increase trend. While previous research has focused solely on either self-repairing coatings or roughness performance, the combination of both has not been thoroughly investigated. Therefore, studying the interlayer adhesion of paint film is vital. Polishing between coatings to enhance the adhesion between each layer of the paint film and sanding the topcoat can make the surface of the paint film smoother, ultimately minimizing the risk of peeling and prolonging the paint film’s lifespan.

The melamine formaldehyde resin, commonly known as melamine resin, is a clear and transparent polymer, which is synthesized by adding melamine and formaldehyde solution to distilled water and then heating and stirring the mixture [14]. This material is primarily utilized as a chemical raw material in manufacturing processes. In addition, shellac is a well-established adhesive, which dries quickly and has been extensively employed in the wood industry [15,16]. The advantages of shellac include its lack of pollutants, non-irritating odor, non-toxicity, and absence of any allergic reactions to the skin. Consequently, it is an appropriate candidate to serve as a core material for microcapsules.

*Tilia* sp. is a wood species, which is resistant to wear and corrosion and is easy to glue and cut, making it suitable for making veneer and plywood [17,18]. Therefore, *Tilia* sp. was chosen as the base material. Meanwhile, the act of grinding wood results in the creation of surface roughness, which in turn enhances the adhesive strength and mechanical durability of coatings [19,20]. The surface roughness of the film and the adhesion existing between its layers have a significant influence on the film’s overall quality [21]. Thus, in this study, self-repairing microcapsules of shellac and water-based paint encapsulated with melamine formaldehyde resin were prepared, and they were added to the water-based paint film to extend the life of the *Tilia* sp. wood coating. The previous study determined that the optimal concentration of microcapsules was 5.0%, which had a certain repair effect [22]. In this research, we investigated the impact of different sandpaper sanding meshes on the optical, mechanical, liquid resistance, aging, and self-repairing properties of *Tilia* sp. films. Sanding between primers increases the adhesion of the paint film, and sanding the topcoat can make the surface of the paint film smoother. This is of vital significance to improve the interlayer adhesion of the coating and optimize the coating structure. The purpose of this study is to explore the effects of different meshes of sandpaper on the optical mechanics and aging resistance of self-repairing coatings and to establish a technical reference for the construction of self-repairing water-based coatings for wooden products.

## 2. Materials and Methods

### 2.1. Materials

The experimental materials and their respective manufacturers are documented in Table 1. Dulux Paint Co., Ltd., Shenzhen, China, provided the Dulux clear base and clear surface coating, which comprise an aqueous acrylic copolymer dispersion, matting agent, additives, and water, with a solid concentration of 30.0%. Guangdong Yihua Technology Co., Ltd., Shantou, China, supplied the *Tilia* sp. with dimensions of 100 mm × 65 mm × 4 mm and 150 mm × 50 mm × 4 mm. The specimens were cut into straight-grain and string-cut panels, with an air-dry density ranging from 0.5 to 0.55 g/cm^3^ at 12.0% moisture content. Distilled water and 75.0% medical ethanol were also utilized. In total, 24 specimens of *Tilia* sp. were prepared and subjected to abrasion using sandpapers of 240 mesh, 400 meshes, 600 mesh, and 1000 mesh.

### 2.2. Preparation of Melamine-Coated Shellac Water-Based Coating Microcapsules

Wall material synthesis: preparing the wall materials, including melamine, formaldehyde, and water, in a mass ratio of 1:2:5. The process began by adding 45.0 g of deionized water to 15.0 g of melamine and 30.0 g of formaldehyde solution, with the pH of the resulting mixture adjusted to 8.0–9.0 using triethanolamine. The solution was then subjected to stirring and heating in a 70 °C water bath using a magnetic stirrer until the wall material reached a state of transparency. Following this, 30.0 g of deionized water was added, and the solution was continuously stirred at 600 rpm for 30 min to obtain the wall material. Finally, the wall material was allowed to cool naturally at room temperature for a period of 10 min.

Preparation of the core material: dissolution of shellac and absolute ethanol in a ratio of 1:5 to create a 20.0% gum solution and isolate impurities through centrifugation. Next, 6.0 g each of Dulux water-based primer and shellac solution were combined in a beaker and uniformly mixed by continuous stirring using a heat-collecting constant temperature heating magnetic stirrer. Then, a solution comprising 107.64 g of distilled water and 0.36 g of sodium dodecylbenzene sulfonate powder was prepared as an emulsifier and added dropwise to the mixture of shellac and water-based paint. The resulting mixture was then stirred in a 70 °C water bath heating magnetic stirrer at a speed of 600 rpm for 60 min to obtain a core material emulsion.

Synthesis of microcapsules: the core material emulsion was subjected to microencapsulation through the gradual addition of melamine resin at a speed of 600 rpm. Then, citric acid was introduced to the mixed solution to adjust the pH value to a range of 2.5–3.0. The mixture was slowly heated to 70 °C and stirred for a duration of 3 h. Upon standing at room temperature for 7 d, a precipitate was generated, which was subsequently washed with deionized water and absolute ethanol and filtered several times with a circulating water vacuum pump. The product obtained was then subjected to heating and drying in an oven at 40 °C for 48 h, resulting in the production of beige powders. These powders comprised shellac water-based paint microcapsules encapsulated with melamine formaldehyde resin.

### 2.3. Preparation of Tilia sp. Self-Repairing Coating

The process of preparing the coating for *Tilia* sp. wood involved storing the wood at a temperature of 23.0 ± 2.0 °C and a relative humidity of 50.0 ± 5.0% for 7 d to achieve a local equilibrium moisture content of 14.9%. Microcapsules were added to water-based paint at different concentrations, and the most effective construction technique for the microcapsules was investigated. The results indicated that the best performance of the *Tilia* sp. paint film was achieved when 5.0% microcapsules were added to the topcoat, with two primers and three topcoats applied. The film showed improved optical, mechanical, aging resistance, and repair properties [23].

**Figure 2:**
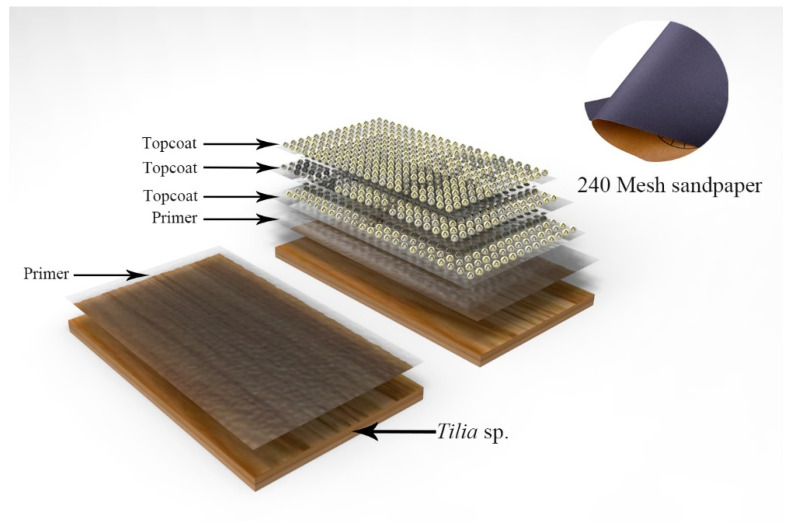
Sanding schematic.

Therefore, an orthogonal test plan was developed to study sanding with different sandpaper meshes (Table 2). The test involved three factors, including sanding between the primers, sanding between the primer/topcoat, and sanding between the topcoats. The sandpaper model used was 240 mesh and 1000 meshes. For example, in test number 1, an example diagram is illustrated in Figure 2. An amount of 2.0 g of the primer was applied to the *Tilia* sp. boards, followed by drying and sanding with 240 mesh sandpaper. The microcapsules, consisting of 0.1 g melamine formaldehyde resin coated shellac water-based paint, were mixed into 1.9 g of water-based topcoat, then painted three times, and polished with 240 mesh sandpaper. The total mass of the paint was 4.0 g, and the coating amount was 0.0616 g/cm^2^.

Based on the orthogonal experiment, an independent test to investigate the impact of sanding mesh on the roughness of the paint film was carried out. The number of sanding meshes between the fixed primer/topcoat was set at 240 mesh, and the number of sanding meshes between the topcoats was set at 1000 mesh. The sandpaper sanding meshes between primers were changed to 0, 240, 400, 600, and 1000 meshes. The experimental design is presented in Table 3. The preparation method used was consistent with the orthogonal test. The brushing process was repeated three times, resulting in a water-based film with a thickness of approximately 100 μm.

### 2.4. Testing and Characterization

#### 2.4.1. Roughness Test of Self-Repairing *Tilia* sp. Paint Films

In accordance with GB/T 1770-2008 [24], the test plate was positioned at the suction cup’s center to enable its adhesion. Subsequently, sandpaper of varying specifications was selected and securely fastened onto the grinding head. To evaluate the roughness of the *Tila* sp. paint film treated with microcapsules and sanded with different mesh sizes, the JB-4C precision roughness tester (manufactured by Shanghai Taiming Optical Instrument Co., Ltd., Shanghai, China) was utilized. The measurement process incorporated a sampling length of 50.0 mm, a measuring tip diameter of 4 µm, and an ISO 2CR filter. Four distinct positions were chosen to be measured.

#### 2.4.2. Optical Properties Test of Self-Repairing *Tilia* sp. Paint Films

As per the specifications outlined in GB/T11186.3-1989 [25], the chromaticity value of a water-based paint film was evaluated using the SEGT-J portable colorimeter (manufactured by Zhuhai Tian-chuang Instrument Co., Ltd., Zhuhai, China). The chromaticity value was measured at the four corners of the paint film. The testing parameters included 45° annular lighting, CIE 10* standard observer, and a CIE D65 light source. The measuring aperture used was 8 mm. The difference in color between the paint film and a standard reference was calculated using Equation (1).

ΔE (color difference) = [(ΔL*)^2^ + (Δa*)^2^ + (Δb*)^2^]^1/2^ (1)

The chromaticity values of the coating film were evaluated by considering various parameters. Specifically, L* represented the brightness, a* indicated the red-green hue, b* represented the yellow-blue hue, C represented the color saturation, and H represented the hue. The chromaticity values of the coating film without microcapsules were denoted by L_1_*, a_1_*, b_1_*, C_1_, and H_1_, while those of the coating film with added microcapsules were denoted by L_2_*, a_2_*, b_2_*, c_2_, and H_2_. The differences in brightness, red-green color, and yellow-blue color between the two films were determined as ΔL* (brightness difference) = L_1_* − L_2_*, Δa* (red-green color difference) = a_1_* − a_2_*, and Δb* (yellow-blue color difference) = b_1_* − b_2_*, respectively.

In order to test the remaining properties of the sample, the sanding process was completed following the independent roughness test. The gloss of the paint film was evaluated using the HG268 smart gloss meter (manufactured by Shenzhen 3nh Technology Co., Ltd., Shenzhen, China), in accordance with the standard outlined in GB/T 4893.6-2013 [26] Test of Surface Coatings of Furniture-Part 6: Determination of Gloss Value. Specifically, the gloss of the paint film was measured at three different angles, namely 20°, 60°, and 85°.

#### 2.4.3. Coating Thickness Test

Conical holes with an apex angle of 120° were deliberately created on the coating under investigation. The walls of these holes were then thoroughly examined using a microscope at 40× magnification. A section of the busbar coating, which is perpendicular to the microscope axis, was selected and inspected for this purpose. To determine the coating thickness, the length of the coated portion of the busbar was precisely measured. It is worth noting that the coating thickness can be determined from the half-length of the busbar coating section based on the principles of trigonometry. Furthermore, the thickness of the three-point water-based topcoat was evaluated using the three-point arithmetic mean method. The findings of this research can significantly contribute to our understanding of the properties and characteristics of the examined coating.

#### 2.4.4. Mechanical Properties Test of Self-Repairing *Tilia* sp. Paint Films

In accordance with ASTM D 882-02 [27], the AG-IC100KN precision electronic universal testing machine (manufactured by Shimadzu, Kyoto, Japan) was employed to determine the tensile strength of the coating. For the purpose of the experiment, a total weight of 4.0 g of the microcapsule coating was brushed onto a glass slide using the method of two primers and three topcoats. After drying, the coating was removed with a knife. The dimensions of the film were 76.5 mm × 25.4 mm, with a thickness of 1.0 mm. The clamp fixing distance was set at 10.0 mm, and the elongation of the paint film was measured as the distance traveled by the clamp until the film broke. The breaking elongation of the paint film was determined using Formula (2).

(2) $$Elongation\;at\;break=\frac{elongation}{10}\times 100\%$$

The hardness, impact resistance, and adhesion of a paint film were assessed using standardized testing methods. The pencil method, as described in GB/T 6739-2006 [28], was utilized to determine the hardness of the paint film. Specifically, a sample plate was placed in a horizontal position, and 6H–6B pencils of varying hardness were gradually applied to the paint film surface at a fixed angle of 45° and a load of 750.0 g until the film exhibited defined defects. The maximum hardness of the pencil before such damage occurred was considered to be the hardness of the paint film.

The QCJ type paint film impactor, in accordance with GB/T 1732-1993 [29], was employed to measure the impact resistance of the paint film. During testing, the paint film of the painted test panel was placed on the anvil, and a heavy hammer weighing 1.0 kg was released from a fixed distance of 50.0 cm from the sliding cylinder onto the film surface. The maximum impact resistance of the coating was recorded when damage to the film occurred.

Additionally, the adhesion of the coating was evaluated using the QFH-HG600 paint film cross-cutting tester, as specified in GB/T 4893.4-2013 [30]. If the intact rate of the coating exceeded 70.0%, it was considered good, whereas anything below this threshold was deemed to be damaged.

#### 2.4.5. Liquid Resistance Test

The method specified in GB/T 1733-93 “Determination of resistance to water of films” [31] was employed to investigate the resistance of a paint film containing microcapsules to various liquids at room temperature. The 15.0% NaCl solution, 70.0% medical ethanol, detergent, and red ink were employed as liquid resistance reagents. To perform the test, the surface of the coating was covered with filter paper soaked in each reagent, followed by a pyrex glass cover, and left undisturbed for a period of 24 h. The chromaticity values and gloss of the paint film were measured both before and after the liquid resistance test.

#### 2.4.6. Aging Test of Self-Repairing *Tilia* sp. Paint Films

During the course of the aging test, *Tilia* sp. boards were subjected to varying conditions with and without microcapsules, containing 5.0% microcapsules at a speed of 600 rpm. These boards were subsequently aged in an oven at temperatures of 100 °C and 150 °C for a duration of 48 h each [32]. The chromaticity values and gloss measurements were recorded at 8 h intervals during the aging process. Furthermore, according to ASTM D4587-2011 [33], the prepared paint film samples were subjected to UV photo-oxidation testing using a UVA-340 fluorescent lamp, with thirty cycles of 8 h irradiation (0.89 W·m^−2^·nm^−1^). An ultraviolet weather-resistant test box manufactured by Nanjing Environmental Testing Equipment Co., Ltd. was utilized to age the boards for 240 h. The UV lamps employed were of the UVA-340 variety, with the UV region being defined as ranging from 290 to 400 nm. The distance between the surface of the sample and the UV lamp was 20.0 mm. The chromaticity values and gloss measurements were conducted every 40 h on the paint films. SEM and infrared spectroscopy analyses were performed on both the *Tilia* sp. paint films prior to and post-aging.

#### 2.4.7. Microstructural and Chemical Composition Analysis

A Quanta-200 scanning electron microscope (SEM, FEI Corporation, Hillsboro, OR, USA) was used to analyze the morphology of the microcapsules. In order to evaluate the self-repairing performance of the paint films, these films were deliberately scratched with a blade of consistent strength. Subsequently, the self-repairing ability of these films was examined at intervals of 7 d using the Zeiss AX10 optical microscope. The surface morphology of the microcapsules as well as the water-based paint film with microcapsules was studied utilizing the Zeiss optical microscope AX10 from Carl Zeiss in Allen, Germany. Additionally, the microcapsule and coating compositions were analyzed via the VERTEX 80 V infrared spectrometer produced by Bruck, Germany, Karlsruhe, Germany. The spectrometer was set to a wavenumber range of 50,000.0–5.0 cm^−1^, signal-to-noise ratio of 55,000:1, and resolution of 0.06 cm^−1^. The chemical compositions of the microcapsules were tested by the KBr tableting, and coatings were examined by ATR tableting.

#### 2.4.8. Coating Formaldehyde Emission Test

According to GB18580-2001 [34], the dry gas method was used to measure the formaldehyde emission of *Tilia* sp. coatings without microcapsules and with 5.0% microcapsules. A glass crystallization dish with a depth of 60 mm containing 300 mL of distilled water was placed at the bottom of a dryer with a capacity of 9–11 L. Then, 10 specimens with dimensions of 150 mm × 50 mm (length × width) were placed in the dryer, and the entire device containing the specimens was finally placed in a (20 ± 2) °C environment for 24 h. At this time, the formaldehyde released from the specimens is absorbed by the distilled water in the crystallization dish, and the formaldehyde release amount of the specimens is determined by detecting the formaldehyde content in the water solution in the crystallization dish.

## 3. Results and Discussion

### 3.1. Microstructure and Composition Analysis of Microcapsules

**Figure 3:**
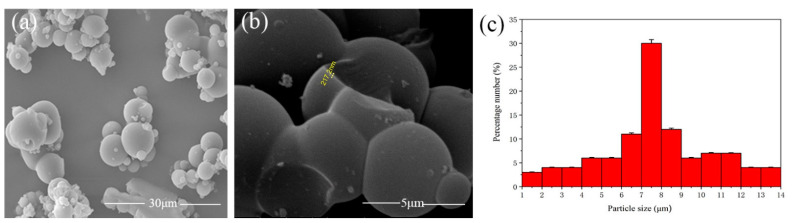
(**a**) SEM images of microcapsules prepared at 600 rpm, (**b**) SEM images of cross-section of microcapsule, (**c**) particle size distribution of microcapsules.

**Figure 4:**
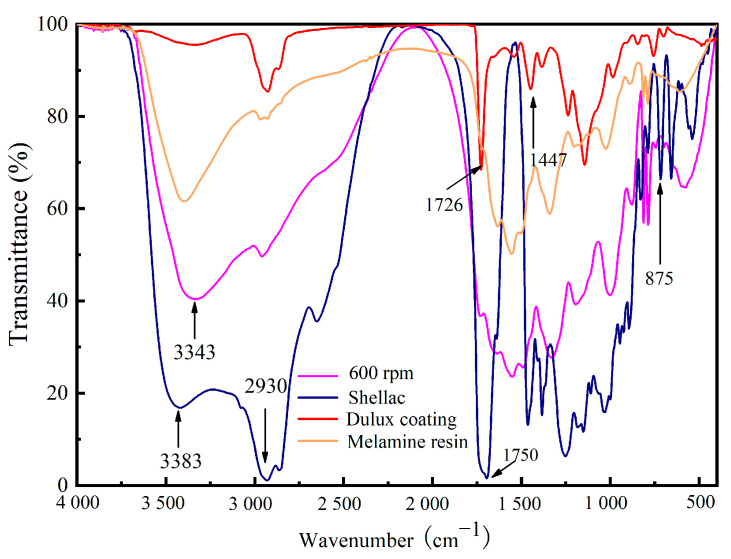
Infrared spectrum of microcapsules.

The scanning electron microscope (SEM) images and particle size distribution map of microcapsules are depicted in Figure 3. Figure 3a displays the various perspectives of microcapsules prepared at 600 revolutions per minute (rpm). Figure 3b shows that the wall thickness of microcapsules is around 200 ± 10 nm. Figure 3c displays the particle size distribution map of microcapsules; 32% of the microcapsules have a particle size between 7 and 8 μm. The microcapsules exhibit a particle size ranging from 3 to 10 microns, with only a few small particle microspheres attached to the spherical microcapsules. Moreover, the majority of the microcapsules are consistent in size. Figure 4 illustrates the infrared (IR) spectrum of the wall and core material composition. Notably, the C–H characteristic absorption peak appears at around 1447 cm^−1^, while 2930 cm^−1^ denotes the characteristic absorptions of –CH_2_ stretching vibration. The 1726 cm^−1^ peak corresponds to the characteristic peak of C=O in water-based paint, and the 3343 cm^−1^ peak signifies the dense NH absorption peak in melamine formaldehyde resin. Furthermore, the 875 cm^−1^ and 3383 cm^−^^1^ peaks reflect the dense –OH absorption peak in the shellac. Notably, the characteristic peaks were observed at the same location, and the peak value of shellac decreased, suggesting the successful encapsulation of the shellac water-based coating by the melamine formaldehyde resin.

### 3.2. Roughness Analysis of Tilia sp. Paint Film

**Figure 5:**
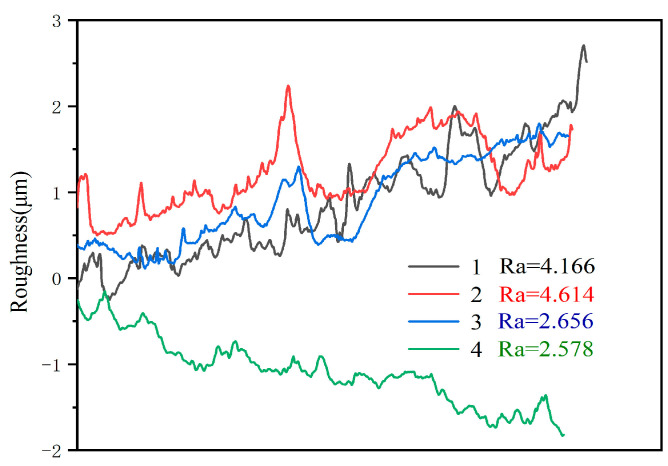
Roughness value of paint film with different sanding meshes.

The results of the roughness of paint film treated with various sandpapers were illustrated in Figure 5. The greater the Ra value, the higher the degree of roughness. Notably, the No. 2 sample displayed the highest level of roughness in polished samples. Similarly, the roughness level of the No. 4 sample was found to be the lowest when treated with 1000 mesh sandpaper between primers and primer/topcoat, and 240 mesh sandpaper between topcoat layers. It is important to note that the findings presented in this study are in line with the understanding of the correlation between sandpaper grit and surface roughness.

The present study employed the range and variance analysis to investigate the roughness value of the paint film. The results of the roughness range analysis, as presented in Table 4 indicated that the range of sanded meshes varied significantly for different primers. The roughness of 240 mesh between the primers of sample No. 1 is 4.2; the roughness of sample No. 2 between the primers is 4.6; and the average value of both is 4.4. The 1000 mesh roughness between the primers of sample No. 1 is 2.7, and the roughness with 1000 mesh between the primers of No. 2 sample is 2.6, with an average value of 2.65. Meanwhile, Table 5 reveals that the variance of the roughness was also affected by the sanding method between the primers. Notably, the largest range and variance of the roughness were observed for the sanded meshes between the primers. Based on the comprehensive analysis of the range and variance of the roughness, it can be inferred that the sanding method between the primers is a significant factor, which affects the roughness of the paint film.

### 3.3. Influence of Different Sandpaper Meshes between Primers on the Optical Properties of Tilia sp. Paint Film

The roughness value was measured after the first coat of the primer was dried and sanded, and the results are presented in Table 6. Sample No. 8, which was sanded with 600 mesh sandpaper between the primers, exhibited the highest roughness value, while sample No. 6, sanded with 240 mesh sandpaper between the primers, showed the smallest roughness value. The findings indicated that the roughness value decreased with the number of sandpaper meshes used between the primers. However, it was observed that sanding with 240 mesh sandpaper between the primers yielded the lowest roughness value, indicating that it is the most suitable sanding method for achieving optimal roughness value of *Tilia* sp. paint film. These results offer valuable insights for the optimization of paint film roughness in the context of primer application.

The color differences between primers sanded with different meshes of sandpaper and the one without sanding were computed and tabulated in Table 7. It was observed that the color difference was maximum when the primer was sanded with 240 mesh sandpaper, while it was minimum when sanded with 1000 mesh sandpaper. The findings also indicated that a smaller sandpaper mesh size resulted in a more pronounced color difference of the primer. These results suggest that the selection of appropriate sanding mesh size is crucial to ensure optimal color match of the primers, particularly in the context of color-sensitive applications.

The effect of sandpaper mesh on the gloss of the primer at different angles, including 20°, 60°, and 85°, with a focus on the gloss value at 60°, was examined. The results showed that the No. 8 sample, which was sanded using 600 mesh sandpaper between the primers, exhibited the highest gloss level (as presented in Table 8), whereas the No. 5 sample, sanded using 0 mesh sandpaper, displayed the lowest gloss value. This can be attributed to the fact that sandpaper with high roughness tends to diminish the surface quality of the paint film, which, in turn, affects the gloss of the paint film. The gloss of the paint film is closely related to the roughness of its surface, whereupon light that strikes the surface is absorbed, reflected, and refracted. The higher the film surface’s smoothness, the greater the reflected light, which results in a higher gloss. Conversely, if the surface of the paint film is uneven, it increases scattered light, leading to a reduction in the primer’s gloss.

### 3.4. Micromorphology and Composition Analysis of Different Sandpaper Meshes between Tilia sp. Primers

**Figure 6:**
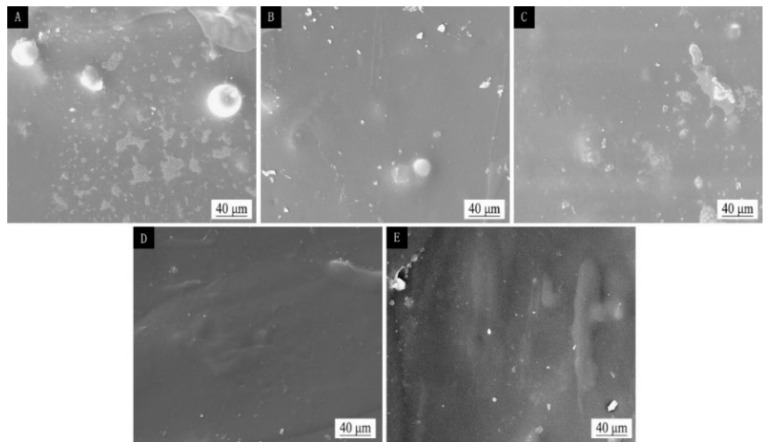
SEM images of different sandpaper meshes between primers, (**A**) 0, (**B**) 240, (**C**) 400, (**D**) 600, (**E**) 1000.

Figure 6 displays the scanning electron microscopy (SEM) images of the primer film sanded with various sandpaper meshes between the primers. As observed, the unsanded primer film was rough, while the primer film sanded with 240 mesh and 400 mesh sandpaper displayed relatively smoother surfaces. The surface of the primer film sanded with 600 mesh sandpaper was slightly uneven, and the surface of the primer film sanded with 1000 mesh sandpaper appeared to be fine sandy and rough. Based on a comprehensive evaluation of the mechanical and optical properties, sanding with 240 mesh sandpaper between the primers resulted in the most favorable outcomes.

### 3.5. Influence of Different Sandpaper Meshes between Primers on the Mechanical Properties of Tilia sp. Paint Film

**Figure 7:**
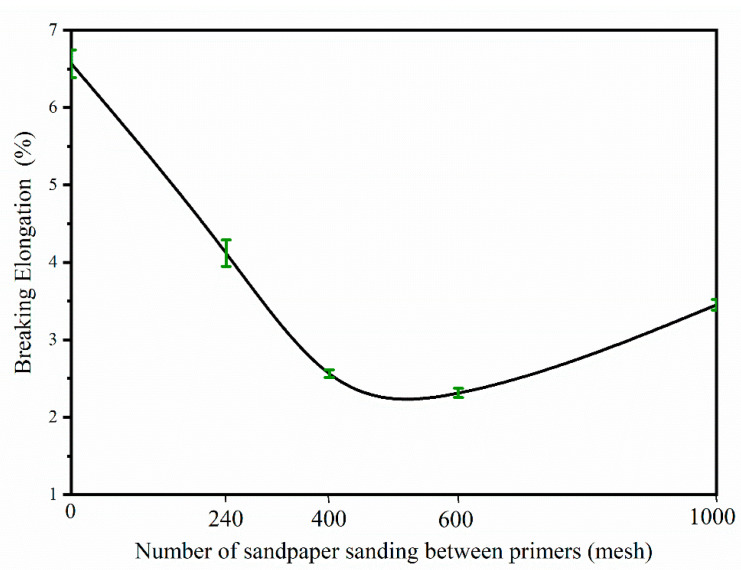
Breaking elongation of paint film with different sandpaper meshes between primers.

Table 9 presents the mechanical properties of the *Tilia* sp. paint film based on in-dependent tests. The paint film exhibited mostly level 2 adhesion, except for the film sanded with 400 mesh sandpaper, which showed level 1 adhesion, and a hardness rating of 5 H. The thickness of the sanded paint film is affected by the size of the sandpaper mesh, with smaller mesh sizes resulting in a thinner film due to increased sanding. The thickness film deviation is approximately ±5.0%. The impact resistance of the paint film without sanding and with 240 mesh sanding was rated level 3, while that of the 400 mesh, 600 mesh, and 1000 mesh sandpaper samples was rated level 2. Therefore, the paint film sanded with 240 mesh sandpaper displayed superior mechanical properties. The breaking elongation of the paint film sanded with different sandpaper meshes between the primers is presented in Figure 7. The highest breaking elongation was observed in the unsanded paint film, followed by the film sanded with 240 mesh sandpaper. Conversely, the breaking elongation of the paint film sanded with 600 mesh sandpaper was the lowest. The number of sandmeshes has little effect on the hardness and impact resistance of the paint film. The substrate mainly affects the hardness of the paint film.

### 3.6. Influence of Different Sandpaper Meshes between Primers on Liquid Resistance of Tilia sp. Paint Film

**Figure 8:**
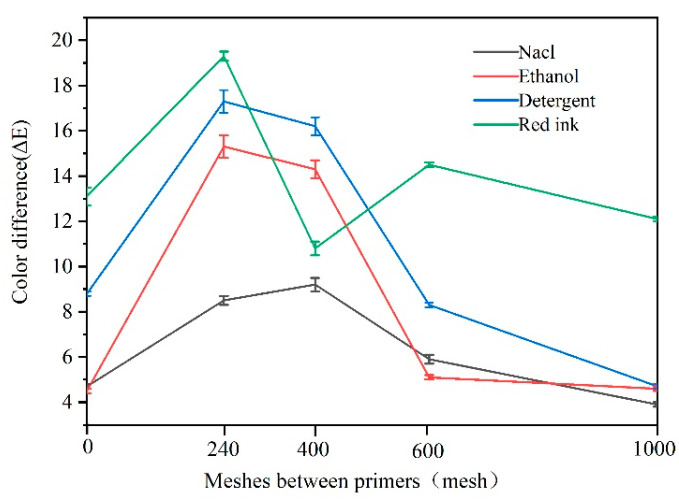
Effect of coating process on color difference of microencapsulated water-based coating after liquid resistance.

**Figure 9:**
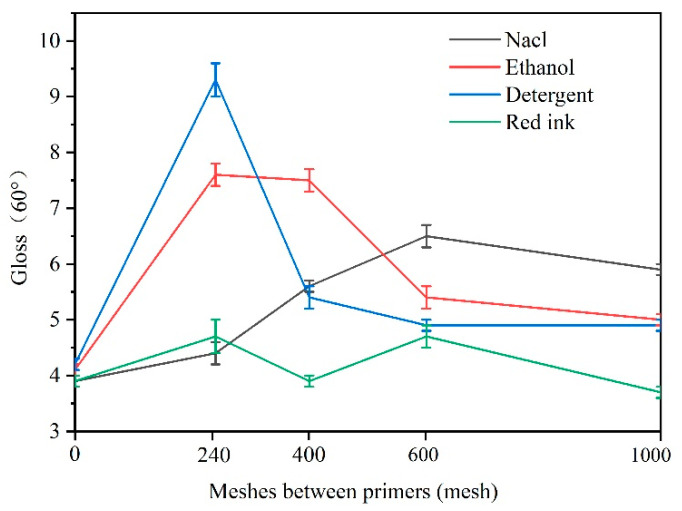
Effect of different sandpaper meshes on 60° gloss difference of *Tilia* sp. paint film after liquid resistance.

Next, we investigated the influence of sandpaper meshes with different sizes on the color and gloss variation of *Tilia* sp. paint film after exposure to various liquid resistance agents. Figure 8 and Figure 9 provide a clear representation of the results obtained. The findings reveal a significant color difference in the *Tilia* sp. paint film after exposure to detergent and red ink, while minimal differences were observed after exposure to NaCl and ethanol.

The color of red ink is red, which mainly affects the red and green values, so the color difference of red ink is the largest. Notably, the use of 240 mesh sandpaper (No. 6 sample) resulted in a considerable variation in both color and gloss. Moreover, the color difference due to liquid resistance decreased as the sandpaper mesh size increased. The rougher the primer, the more pronounced the concavity and convexity of surface particles, as well as the greater the specific surface area of the particles, resulting in an increased liquid absorption area. Based on the results, the *Tilia* sp. paint film’s liquid resistance rating was classified as level 1 after being exposed to NaCl, ethanol, and detergents, while it was graded as level 2 following exposure to red ink. For the balance between the color difference and gloss of the paint film, the gloss is more valuable in the case of adding microcapsules. It is ideal to polish the primer with 240 mesh sandpaper.

### 3.7. Influence of Different Sandpaper Meshes between Primers on Aging Resistance and Self-Repairing Performance of Tilia sp. Paint Film

**Figure 10:**
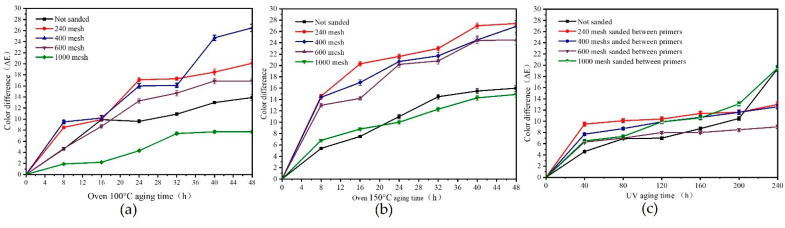
Effect of different environment on color difference of *Tilia* sp. film with different sandpaper meshes between primers, (**a**) oven 100 °C aging, (**b**) oven 150 °C aging, (**c**) UV aging.

Figure 10 demonstrates the impact of aging time on the color difference of *Tilia* sp. paint film with various sandpaper mesh sizes. For the *Tilia* sp. paint film aged at 100 °C, the color difference gradually increases with longer aging time. Among the samples sanded with different mesh sizes, the paint film sanded with 1000 mesh showed the smallest color difference, followed by the unsanded paint film. The highest color difference was observed for the paint film sanded with 400 mesh sandpaper after 48 h of aging. Similarly, for the *Tilia* sp. paint film aged at 150 °C, the color difference increased as the aging time was prolonged. The smallest color difference was observed for the paint film sanded with 1000 mesh sandpaper, followed by the unsanded paint film. The color difference of the paint film sanded with other sandpaper meshes was not significantly different. After 48 h of aging at 150 °C, the *Tilia* sp. film undergoes carbonization, and the paint film ages due to internal and external stress. In addition, the color difference of the UV-aged *Tilia* sp. paint film also increases over time. After 240 h of UV aging, the paint film sanded with 240 mesh sandpaper exhibited a stable color difference. This is because ultraviolet radiation causes solvent volatilization, leading to paint film weight loss. When water-based paints were applied to coat *Tilia* sp. with UV aging, the UV light decomposed the lignin in the wood, resulting in reduced adhesion of the paint film to the wood surface and alteration of the color of the painted wood. The uneven adhesion between the layers caused by different roughness results in uneven aging color difference. The 1000 mesh sandpaper is more smooth, and the surface of the paint film is more uniform, so the color difference is small.

**Figure 11:**
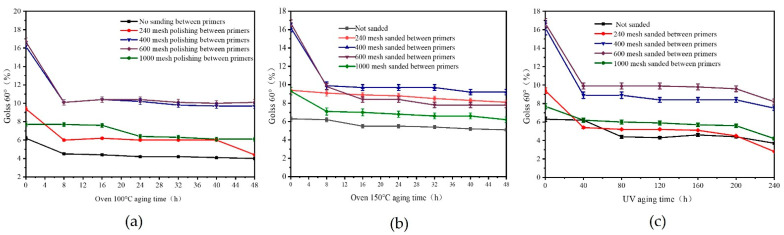
Effect of different environment on gloss of *Tilia* sp. film with different sandpaper meshes between primers, (**a**) oven 100 °C aging, (**b**) oven 150 °C aging, (**c**) UV aging.

Figure 11 presents the findings of the impact of aging time on the 60° gloss of the *Tilia* sp. paint film with various sandpaper meshes. The results indicated that, as the aging time was extended during the aging process at 100 °C and 150 °C, the gloss of the *Tilia* sp. paint film gradually decreased and eventually stabilized. Subsequently, the gloss of the *Tilia* sp. paint film significantly reduced after 40 h of UV aging and gradually returned to a smooth state. However, the gloss showed a declining trend again after 200 h of aging, and the 400/600 mesh sandpaper showed the highest impact of gloss on aging. The structure of each layer is different, resulting in irregular gloss changes. Overall, the anti-aging gloss of the *Tilia* sp. paint film sanded with 240 mesh sandpaper between the primers was relatively stable.

**Figure 12:**
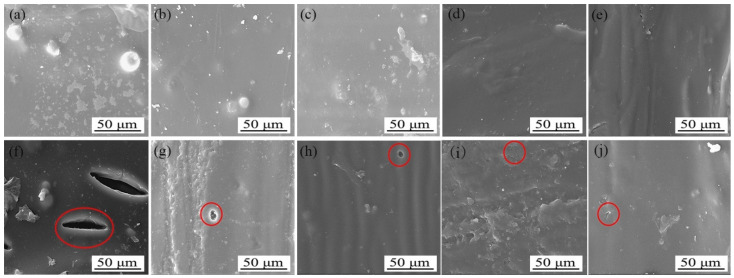
SEM of *Tilia* sp. film with different sandpaper meshes before and after 100° C aging, before aging: (**a**) 0, (**b**) 240, (**c**) 400, (**d**) 600, (**e**) 1000; after aging: (**f**) 0, (**g**) 240, (**h**) 400, (**i**) 600, (**j**) 1000.

**Figure 13:**
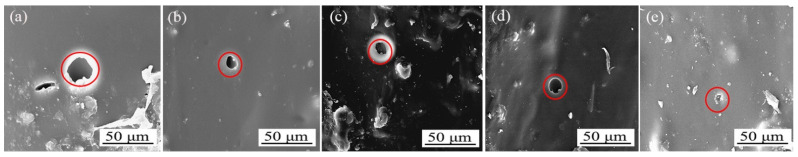
SEM of *Tilia* sp. film with different sandpaper meshes after 150° C aging: (**a**) 0, (**b**) 240, (**c**) 400, (**d**) 600, (**e**) 1000.

**Figure 14:**
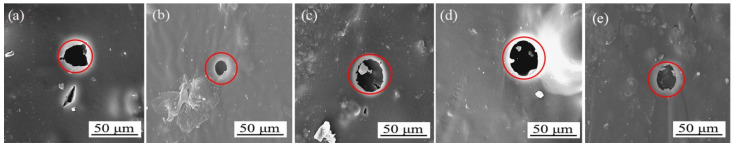
SEM of *Tilia* sp. film with different sandpaper meshes after UV aging: (**a**) 0, (**b**) 240, (**c**) 400, (**d**) 600, (**e**) 1000.

The influence of aging on the *Tilia* sp. paint film was investigated by analyzing scanning electron microscope (SEM) images taken before and after aging at 100 °C, 150 °C, and UV aging, with and without sanding with 240 mesh between the primers. Scanning electron microscope (SEM) images were taken before and after aging at 100 °C, 150 °C, and UV aging, as shown in Figure 12, Figure 13 and Figure 14. Figure 12a represents the unsanded *Tilia* sp. paint film before aging, while Figure 12b represents the sanded *Tilia* sp. paint film using 240 mesh between the primers before aging. Figure 12c–e show the 100 °C aging effect of paint film sanded with 400, 600, and 1000 mesh sandpaper. The addition of microcapsules to the topcoat caused the surface of the paint film to appear grainy and not smooth. SEM images showed that the unsanded *Tilia* sp. paint film exhibited obvious bubble after being aged in an oven at 100 °C, as shown in Figure 12f. On the other hand, the paint film with 240 mesh sanded between the primers and after aging in an oven at 100 °C resulted in irregular circular cracks, as shown in Figure 12g, as well as Figure 12h–j. Figure 13a shows the morphology of the unsanded *Tilia* sp. paint film after aging in an oven at 150 °C, with large irregular shaped cracks. Figure 13b shows the morphology of the 240 mesh sanded paint film between the primers after being aged at 150 °C in an oven, which also resulted in small circular cracks. Figure 14a shows the morphology of the unsanded *Tilia* sp. paint film after UV aging, which produced irregular circular cracks. Figure 14b shows the morphology of the 240 mesh sanded paint film between the primers after UV aging, which produced round cracks. Cracks also appeared in other meshes. The cracks produced by the *Tilia* sp. paint film with microcapsules were relatively regular and small after aging, indicating that the sanding meshes have a retarding effect on cracks. This can be attributed to the adhesion effect of sanding on the stability of the paint film, improving its quality. During the aging process, the wall material of the microcapsule cracked, and the core material flowed out, gradually mending the cracks and reducing their size. After UV aging, the paint film cracks and blisters appear in different degrees. The 240 mesh sanded paint film has smaller circular cracks after aging, which is due to better intercoat adhesion. Therefore, the primer is sanded with 240 mesh sandpaper, and the aging effect is the best.

**Figure 15:**
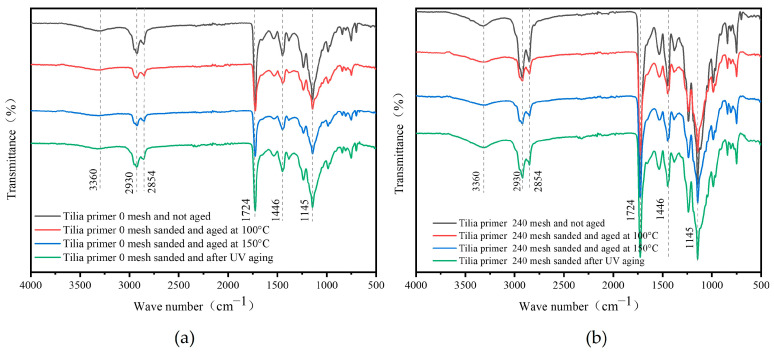
Infrared spectrum of *Tilia* sp. paint film before and after aging with different sandpaper meshes between primers: (**a**) 0, (**b**) 240 mesh.

Figure 15 presents the infrared spectra of the *Tilia* sp. Paint film before and after aging, with and without 240 mesh sanding between the primers. The transmittance of *Tilia* sp. film after UV aging was decreased compared to that of temperature aging, and this difference was noticeable. Simultaneously, the role of sandpaper and loss of microcapsules led to a decline in the absorption of infrared bands, resulting in a decrease in the 240 mesh film compared to unsanded film. The stretching vibration peaks of –CH_2_, 2930 cm^−1^ and 2854 cm^−1^, and 1450 cm^−1^, respectively, and the characteristic peak of C=O in water-based coatings, 1727 cm^−1^, were observed. Additionally, the N-H absorption peak in the wall material melamine resin was observed at 3360 cm^−1^. No peak disappeared or appeared before and after aging, indicating that the composition of the *Tilia* sp. paint film did not differ before and after aging.

**Figure 16:**
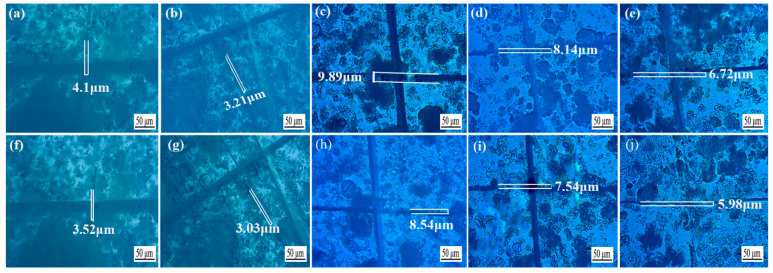
Microscope pictures of *Tilia* sp. film before and after repairing with different sandpaper meshes between primers. Before repairing, (**a**) 0 mesh, (**b**) 240 mesh,(**c**) 400 mesh, (**d**) 600 mesh, (**e**) 1000 mesh; after repairing, (**f**) 0 mesh (**g**) 240 mesh,(**h**) 400 mesh, (**i**) 600 mesh, (**j**) 1000 mesh.

**Figure 17:**
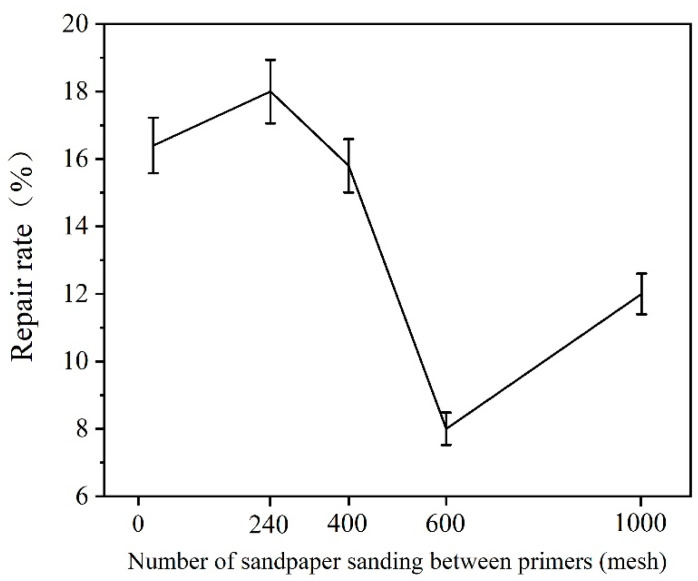
Repair rate of paint film with different sandpaper meshes between primers.

The *Tilia* sp. paint films that were not sanded between the primers and sanded with 240, 400, 600, 1000 mesh sandpaper were compared by scratching with a blade to observe the repair effect after 7 d. The results of the observations are presented in Figure 16, and the repair rate of paint films with different sandpaper meshes between the primers is displayed in Figure 17. The scratch of the unsanded primer was repaired from Figure 17a 4.1 ± 0.21 μm to Figure 17f 3.52 ± 0.18 μm; the repair rate reached 16.0%. The scratch of the sanded primer with 240 mesh sandpaper was repaired from Figure 17b 3.21 ± 0.16 μm to Figure 17g 3.03 ± 0.15 μm; the repair rate was about 18.0%. The scratch repair rates of primers sanded with 400, 600, and 1000 mesh sandpaper were 15.8%, 8.0%, and 12.0%, respectively. These results indicated that microcapsules had a certain repair effect. The scratches on the paint film sanded with 240 mesh sandpaper between the primers have the highest repair efficiency.

The curing mechanism of water-based acrylic coatings involves bulk curing, which occurs under the influence of heat. In the artificial scratch repair test, the shellac curing mechanism is initiated by cracks that form under the influence of stress, with the core material overflowing. The solvents in both water-based acrylic paints and shellac evaporate and drain through pores in the coating. Subsequently, water-based acrylic acid undergoes a cross-linking reaction, and shellac is physically cured to fill the microcracks, thereby achieving a self-repairing effect.

### 3.8. Formaldehyde Emission

The formaldehyde emission from *Tilia* sp. treated with a 5.0% microcapsule content was found to be 0.3 mg/L (Table 10), which falls well below the minimum threshold required by the national standard. During the process of microcapsule preparation, the amount of free formaldehyde gradually diminishes. As per Stéphane Bône [35], melamine resin is a type of thermosetting resin, which undergoes polymerization through the use of melamine and formaldehyde. This study reports a molar ratio of 1:3 for melamine to formaldehyde. In addition, melamine was found to be an effective means of substantially decreasing the level of free formaldehyde in the slurry.

## 4. Conclusions

An orthogonal experiment revealed that sanding between the primers had the most significant effect. Furthermore, an independent experiment showed that increasing the mesh size resulted in decreased surface roughness and decreased color difference, elongation at break, and gloss after liquid resistance. When the primer is sanded with 240 mesh, the elongation at break is relatively high. Additionally, the color difference of the paint film increases with the aging time. The anti-aging gloss of 240 mesh sandpaper used between the primers remained relatively stable. The paint film sanded with 240 mesh sandpaper between the primers displayed small and regular circular cracks after temperature and UV aging. From the perspective of repair efficiency, the paint film repair effect obtained by sanding with 240 mesh sandpaper is the best. Overall, the paint film demonstrated good comprehensive performance when sanded with 240 mesh between the primers, 240 mesh between the primer/topcoat, and 1000 mesh for the topcoat. Self-repairing microcapsules showed better repair efficacy on the coating. This coating technology offers a crucial technical method for self-repairing coating wood products.

## Figures and Tables

**Table 1:** Experimental materials.

Raw Material	Molecular Weight	CAS	Manufacturers
The 37.0% formaldehyde solution	30.03	50-00-0	Dongjiang Reaoent, Binzhou, China.
Citric acidmonohydrate	210.14	5949-29-1	Shanghai Aladdin Biochemical Technology Co., Ltd., Shanghai, China.
Melamine	126.12	203-615-4	Zhejiang Longyou Sihai Chemical Co., Ltd., Quzhou, China.
Triethanolamine	149.1882	102-71-6	Jinnan Kaijun Chemical Co., Ltd., Jinan, China.
Ethyl acetate	88.11	141-78-6	Henan Chun Ao Shi hua Technology Co., Ltd., Zhenzhou, China.
Dodecyl benzene sulfonate sodium	348.476	25155-30-0	Shanghai Honsun Biological Technology Co., Ltd., Shanghai, China.
Anhydrous ethanol	46.07	64-17-5	San He Shun Chemical Co., Ltd., Wuhan, China.
Shellac film (the compositions of shellac are shellac resin, shellac pigment, shellac wax, sugar, protein, etc.)			Jinan Dahui Chemical Technology Co., Ltd., Jinan China.
15.0% NaCl solution			Su Yan Group Co., Ltd., Nanjing, China.
Liby detergent			Liby Science and Technology Co., Ltd., Guangzhou, China.
Red ink			Shanghai Fine Stationery Co., Ltd., Shanghai, China.

**Table 2:** L2^3^ orthogonal experiment plan for different sanding meshes.

Test Number	Sanding between Primers	Sanding between Primers/Topcoats	Sanding between Topcoats
1	240	240	240
2	240	1000	1000
3	1000	240	1000
4	1000	1000	240

**Table 3:** Independent experiment for sandpaper sanding.

Test Number	Sanding between Primers	Sanding between Primers/Topcoats	Sanding between Topcoats
5	0	240	1000
6	240	240	1000
7	400	240	1000
8	600	240	1000
9	1000	240	1000

**Table 4:** Range analysis of film roughness sanded with different sandpaper meshes.

Factor	Sanding between Primers	Sanding between Primer/Topcoat	Sanding between Topcoats	Roughness
1	240	240	240	4.2
2	240	1000	1000	4.6
3	1000	240	1000	2.7
4	1000	1000	240	2.6
Mean value 1	4.4	3.45	3.45	
Mean value 2	2.65	3.6	3.65	
Range	1.75	0.15	0.2	

**Table 5:** Variance analysis of film roughness sanded with different sandpaper meshes.

Factor	Error Square Sum	Degree of Freedom	F Ratio	F Critical Value	Significance
Between primers	3.1	1	2.9	10.1	
Between primers/topcoats	0	1	0	10.1	
Sanding between topcoats	0.1	1	0.1	10.1	
Variance	3.3	3			

**Table 6:** Roughness value of *Tilia* sp. primer sanded with different sandpaper meshes.

Test Number	Ra (um)
5	3.3 ± 0.1
6	3.2 ± 0.1
7	3.1 ± 0.1
8	2.9 ± 0.1
9	2.5 ± 0.1

**Table 7:** Colorimetric values and color differences of *Tilia* sp. paint film in independent experiment.

Test Number	L	a	b	c	H	ΔE
5	60.3 ± 1.8	17.7 ± 0.3	30.6 ± 0.6	31.6 ± 1.0	54.5 ± 1.8	-
6	75.2 ± 2.0	10.2 ± 0.4	29.7 ± 0.8	31.4 ± 0.8	71.0 ± 1.4	16.7 ± 0.2
7	72.1 ± 3.1	11.1 ± 0.1	29.5 ± 0.5	31.6 ± 1.0	69.2 ± 1.2	13.6 ± 0.1
8	69.4 ± 0.6	13.6 ± 0.1	27.4 ± 0.6	30.6 ± 0.4	63.6 ± 1.0	10.5 ± 0.4
9	60.0 ± 1.9	15.9 ± 0.4	28.2 ± 0.3	32.4 ± 0.5	60.6 ± 0.4	3.0 ± 0.1

**Table 8:** Gloss of *Tilia* sp. paint film in independent experiment.

Test Number	Gloss (%)		
	20°	60°	85°
5	2.3 ± 0.1	6.1 ± 0.1	3.2 ± 0.1
6	2.4 ± 0.1	7.9 ± 0.1	5.5 ± 0.1
7	2.7 ± 0.1	13.1 ± 0.2	9.3 ± 0.1
8	4.0 ± 0.1	16.2 ± 0.2	17.9 ± 0.4
9	4.6 ± 0.1	16.5 ± 0.2	26.4 ± 0.8

**Table 9:** Independent experiment on mechanical properties of *Tilia* sp. film.

Test Number	Adhesion (Level)	Hardness	Impact Resistance (N/mm)
5	2	5H	3
6	2	5H	3
7	1	5H	2
8	2	5H	2
9	2	5H	2

**Table 10:** Formaldehyde emission of *Tilia* sp. coating.

Microcapsule Concentration	Formaldehyde Emission (mg/L)	Standard (mg/L)
0	0.1	≤1.5
5.0%	0.3	≤1.5

## Data Availability

All the data are available within the manuscript.
